# Development of bile duct bezoars following cholecystectomy caused by choledochoduodenal fistula formation: a case report

**DOI:** 10.1186/1471-230X-6-1

**Published:** 2006-01-05

**Authors:** Jamal Akhavan Moghaddam, Mohsen Amini, Soheil Adibnejad

**Affiliations:** 1 Department of General Surgery, Baqiyatallah Hospital, Baqiyatallah University of Medical Sciences, Mollasadra Street, Tehran, Iran; 2 Department of Gastroenterology, Baqiyatallah Hospital, Baqiyatallah University of Medical Sciences, Mollasadra Street, Tehran, Iran

## Abstract

**Background:**

The formation of bile duct bezoars is a rare event. Its occurrence when there is no history of choledochoenteric anastomosis or duodenal diverticulum constitutes an extremely scarce finding.

**Case presentation:**

We present a case of obstructive jaundice, caused by the concretion of enteric material (bezoars) in the common bile duct following choledochoduodenal fistula development. Six years after cholecystectomy, a 60-year-old female presented with abdominal pain and jaundice. Endoscopic retrograde cholangiopancreatography demonstrated multiple filling defects in her biliary tract. The size of the obstructing objects necessitated surgical retrieval of the stones. A histological assessment of the objects revealed fibrinoid materials with some cellular debris. Post-operative T-tube cholangiography (9 days after the operation) illustrated an open bile duct without any filling defects. Surprisingly, a relatively long choledochoduodenal fistula was detected. The fistula formation was assumed to have led to the development of the bile duct bezoar.

**Conclusion:**

Bezoar formation within the bile duct should be taken into consideration as a differential diagnosis, which can alter treatment modalities from surgery to less invasive methods such as more intra-ERCP efforts. Suspicions of the presence of bezoars are strengthened by the detection of a biliary enteric fistula through endoscopic retrograde cholangiopancreatography. Furthermore, patients at a higher risk of fistula formation should undergo a thorough ERCP in case there is a biliodigestive fistula having developed spontaneously.

## Background

The recurrence of obstructive jaundice after cholecystectomy is estimated to occur in one to seven per cent of all cholecystectomy cases [1-4]. Symptoms may be due to retained stones which were unrecognized at the time of the initial operation, the development of a bile duct stricture or the presence of a long cystic duct remnant. Furthermore, parasitic infections of the hepatobiliary system, such as fascioliasis and ascariasis, account for other rare causes of recurrent bile duct obstructions, which may mimic the choledocholithiasis picture [5-9].

We present another cause of cholestatic jaundice, not commonly considered in differential diagnoses: the obstruction of the extrahepatic bile ducts by the concretions of fibrinoid materials (biliary bezoars), attributed to the existence of a fistula between the duodenum and the bile duct.

## Case presentation

The patient is a 60-year-old Iranian female referred to our hospital with several-weeks history of abdominal pain, severe pruritus, yellow sclera, light-colored stool, and nausea and vomiting. In addition to fever and chills, she reported intermittent episodes of sustained abdominal pain in the past two months, which worsened by meals and radiating to the right-scapular area. Six years previously, she had undergone cholecystectomy without choledochoenteric anastomosis for symptomatic cholelithiasis.

The patient was a known case of rheumatoid arthritis and diabetes mellitus, her medications consisting of insulin, prednisolone, chloroquine and methotroxate.

On admission, the patient had a temperature of 38.5 C and was noted to have icteric sclera. A right upper quadrant scar was seen on her abdomen. The patient's abdomen was soft and non-distended with normal bowel sounds, albeit tender to deep palpation in the epigastrium and the right upper quadrant. There was no evidence of rebound tenderness or peritoneal signs.

Laboratory data on admission were notable for an elevated total bilirubin of 7.1 mg/dl (nl 0.2–1.1); direct bilirubin of 4.7 mg/dl (nl up to 0.5); alkaline phosphatase of 1217 IU/L (nl 64–306); SGOT of 78 U/L (nl 5–40); and SGPT of 185 U/L (nl 5–40). The total white blood cell count was 10,800 /cmm; prothrombin time was 13 second (nl 10–13) and the c-reactive protein was 2+.

**Figure 1:**
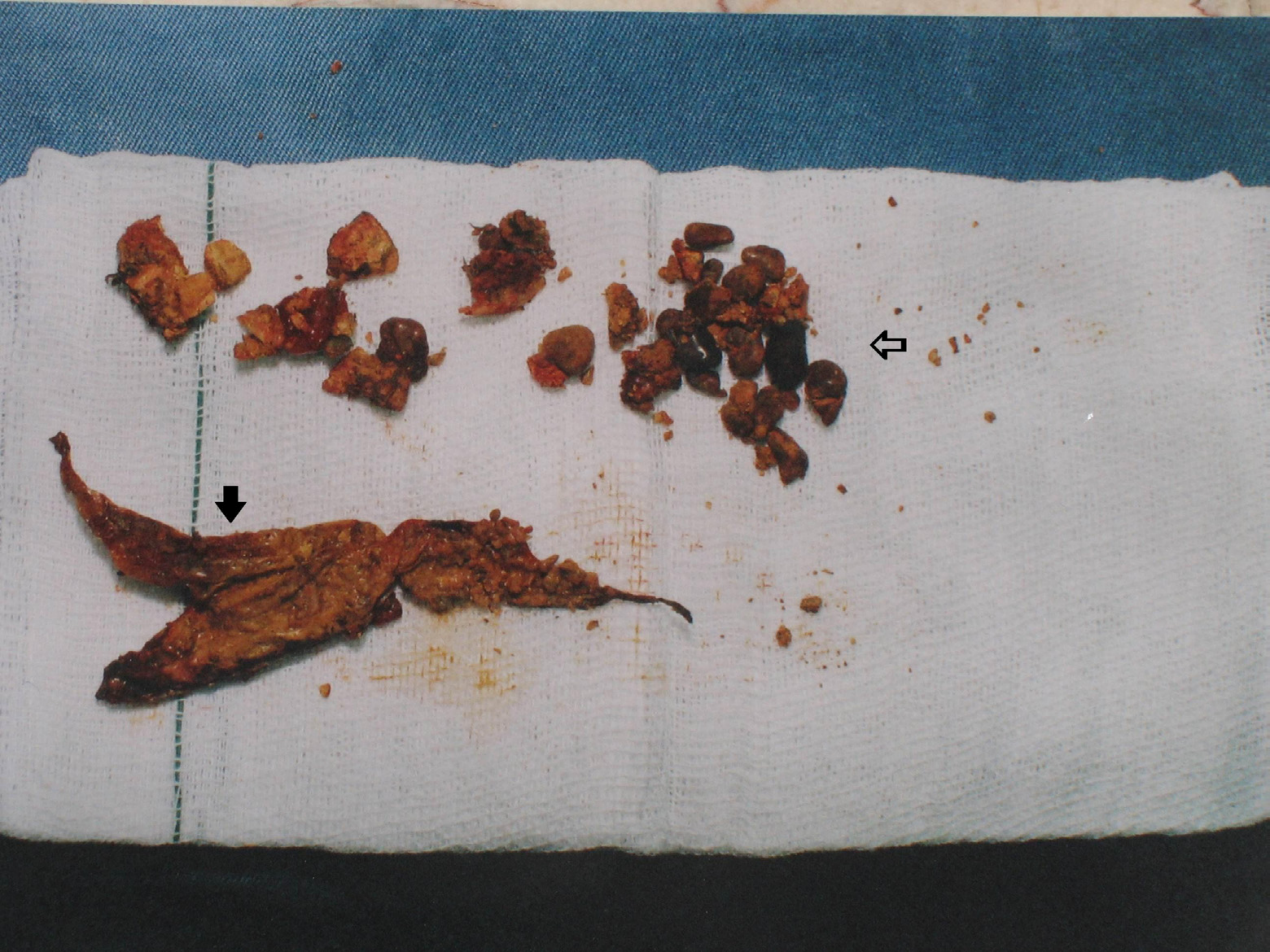
**Obstructing materials**. Bezoar material in the shape of a CBD cast (solid arrow) and multiple stones (open arrow) removed from the dilated common bile duct by surgery.

**Figure 2:**
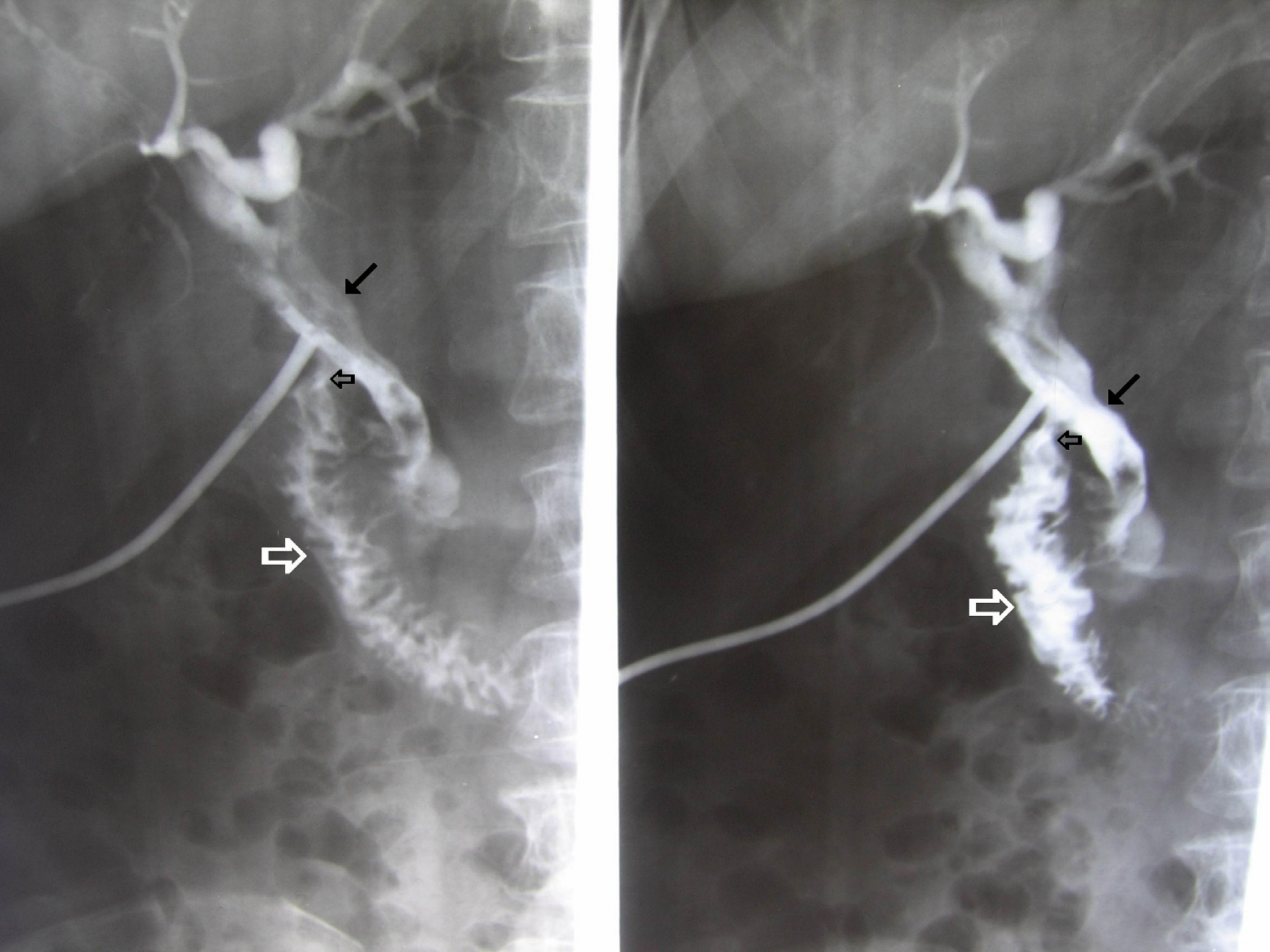
**The post-operative T- tube cholangiography**. It is demonstrating a fistula (open black arrow) located between the CBD (solid arrow) and the duodenum (open white arrow).

Abdominal ultrasound demonstrated the dilation of the biliary tree with visible choledocholithiasis. Abdominal CT-scan revealed a dilated biliary tract with air entrapment in the lateral side of the right lobe of the liver. The patient subsequently underwent endoscopic retrograde cholangiopancreatography (ERCP). The ERCP illustrated multiple stones, the prominent one of which was a 2.5-cm mass. Despite attempts for lithotripsy and sphincterotomy, however, ERCP failed to retrieve all the materials. Therefore, the remaining stone- like objects and materials were successfully removed by means of common bile duct (CBD) exploration (Figure 1). Intra-operative findings included multiple stones and a 2.5-cm nonhomogenous mass, impacted in a dilated CBD and having a distorted anatomy due to adhesions, without any signs of previous anastomoses. A drainage T-tube was inserted through CBD. Nine days after the surgery, a post-operative T-tube cholangiography accidentally detected a long choledochoduodenal fistula (Figure 2). Since the whole management process from ERCP to T-tube cholangiography did not take more than 3 weeks, both the endoscopist and the surgeon must have failed to detect the fistula because of its overlap with the duodenum and the distorted anatomy.

A histological assessment of the objects removed revealed fibrinoid materials with some cellular debris. The patient remained well during the one-year follow-up.

## Discussion

This report describes a case of cholestatic jaundice caused by concretions of fibrinoid materials obstructing the extrahepatic bile ducts (biliary bezoars). It is noteworthy that there is a paucity of information regarding the formation of the biliary tract bezoar.

The first case report of bezoar-induced obstructive jaundice dates back to 1989, in which Seyrig JA et al. reported a case of cholestasis caused by an intradiverticular bezoar [10]. The second case report (1993) is about a brown pigment gallstone, having formed around a phytobezoar without spontaneous biliary enteric fistula [11]. Lamotte M et al. (1995) described the development of a biliary phytobezoar 15 years after surgical cholecystogastrostomy [12]. In another study, gallstones containing surgical suturing material were found in a number of patients who had undergone a previous cholecystectomy [13].

The formation of the fistula, detected accidentally through post-operative cholangiography, is believed to be the cause of bezoar development in the present study.

## Conclusion

Induced by intra-enteric materials transmitted through long fistulae, bezoar formation should be considered as an extremely rare differential diagnosis for obstructive jaundice since it is not normally associated with such relatively common predisposing factors as choledochoenteric anastomosis or duodenal diverticulum The ability of ERCP in detecting fistulae in a case of obstructive jaundice helps less invasive measures (more intra-ERCP views and efforts) outweigh surgery. The detection of biliodigestive fistulae in obstructive jaundice patients requires a thorough ERCP. Moreover, when the macroscopic characteristics of the stones resemble those of bezoar material, it is expedient that surgeons allocate more time and effort to detecting and ligating the possible fistula. We hope that this case report of fistula formation with subsequent bezoar production and obstructive jaundice will raise the awareness of gastroenterologists and surgeons about such an interesting phenomenon.

## Competing interests

The author(s) declare that they have no competing interests.

## Authors' contributions

JAM: Contributed to surgical management and the final scientific revision of manuscript.

MA: Performed ERCP and assisted in preparation of the draft manuscript and the final revision of manuscript.

SA: Contributed to the preparation of manuscript and literature review.

All the authors were involved in the management of the patient and approved the final manuscript.

## Pre-publication history

The pre-publication history for this paper can be accessed here:
